# Discrete early maladaptive schema subgroups in remitted bipolar disorders: association with neuropsychological performance, residual symptoms, and psychosocial functioning

**DOI:** 10.3389/fpsyg.2025.1476345

**Published:** 2025-03-18

**Authors:** Katia M’Bailara, Caroline Munuera, François Weil, Christine Passerieux, Paul Roux

**Affiliations:** ^1^University of Bordeaux, LabPsy, Bordeaux, France; ^2^Réseau des Centres Expert des Troubles Bipolaires, Fondation FondaMental, Créteil, France; ^3^Pôle PGU, Centre Hospitalier Charles Perrens, Bordeaux, France; ^4^Centre Hospitalier de Versailles, Service Universitaire de Psychiatrie d’Adulte et d’Addictologie, Le Chesnay, France; ^5^Faculté de Médecine Paris-Saclay - Université Paris-Saclay, Le Kremlin Bicetre, France; ^6^UFR Simone Veil Santé, Université de Versailles Saint-Quentin-En-Yvelines, Montigny-le-Bretonneux, France; ^7^DisAP - Moods - CESP, INSERM UMR1018, Villejuif, France

**Keywords:** bipolar disorders, euthymia, early maladaptive schemas (EMS), psychosocial functioning, residual symptoms, neuropsychological performance, clustering

## Abstract

**Objectives:**

To better understand the disability and heterogeneity in terms of residual symptoms and psychosocial and cognitive functioning in bipolar disorders (BD), individual discrepancies in the activation of early maladaptive schemas (EMS) are relevant to investigate. This study aimed to identify activation profiles of EMS and to investigate the association between identified profiles and disability during euthymia.

**Design:**

This is a cross-sectional study.

**Methods:**

Clinical data, psychosocial functioning, neuropsychological performance and EMS were collected in euthymic outpatients with a BD. Clustering was performed on EMS activation, followed by inter-cluster comparisons on variables above using *post-hoc* tests. A multivariate regression was used to confirm associations between clusters and variables of interest by controlling for covariates.

**Results:**

Thanks to a person-oriented approach, our results showed three profiles of EMS: “Hypoactivation,” “Light activation.” and “Major Hyperactivation.” Individuals in the light and major hyper activated clusters had worse psychosocial functioning compared to individuals in the hypoactivated cluster. There were no differences in neuropsychological performance between the different profiles of EMS, thus suggesting the independence of these sources of variance in psychosocial functioning of individuals with BD.

**Conclusion:**

This paper highlights the importance of considering individual personality and functioning to better understand the heterogeneity in BD during euthymia. For some people, schema therapy seems particularly relevant due to the overactivation of EMS, and even more so because these people have particularly marked functional impairments and clinical severity.

## 1 Introduction

Bipolar disorder (BD) is a severe and persistant illness with high impact, even outside of manic or depressive episodes, ranked second in days out of role per year (Alonso et al., 2011). A key issue in clinical practice is to identify personal factors associated with disability level (Rosa et al., 2007; Grande et al., 2013). Firstly, individuals with BD report a large number of residual symptoms, such as emotional dysregulation, disruption of circadian rhythms, cognitive complaints, guilt, low self-esteem and physical pain (Samalin et al., 2014). Secondly, even in the euthymic phase, people also report a high level of functional impairment (Samalin et al., 2014). There is not a single profile of functioning in individuals with BD (Roux et al., 2017), highlighting heterogeneity in functioning during the euthymic period. It now appears crucial to understand what might explain such differences. Impaired functioning is closely related to residual depressive symptoms (Fagiolini et al., 2005) and to deficits in neuropsychological performance (Roux et al., 2017; Léda-Rêgo et al., 2020).

Moreover, cognitive impairments are recurrent claims of people during euthymic phases. Subjective cognitive problems are reported by 2/3 of people with BD, particularly memory problems, attentional problems, and executive function problems (Martínez-Arán et al., 2005). Objective neuropsychological impairments affect key cognitive dimensions, such as verbal memory, attention, processing speed, executive functions (Bourne et al., 2013), social cognition with small to moderate deficits in theory of mind, small deficits in emotional recognition (Samamé et al., 2015), and risky decision making (Gorlyn et al., 2013). However, there is wide variation in the estimated prevalence of these deficits, ranging from 12.4% (Roux et al., 2019) to 34% (Tsapekos et al., 2021), or even 57.7% (Eric et al., 2013). Some bipolar patients have global impairment, some having selective moderate impairment, and others have good cognitive performance (Roux et al., 2017; Green et al., 2020). Several determinants of these neuropsychological deficits have been explored to understand this heterogeneity but data remain insufficient. There is no consensus on the relationship between cognitive functioning and residual thymic symptoms. Residual depressive symptoms have been reported to have a negative effect on cognitive functioning in some studies (Bourne et al., 2013), but not in others (Cullen et al., 2016; Roux et al., 2017).

It becomes important to better understand the basis of such a range of cognitive and functional impairments. Previous work has shown that personality is an important determinant of functioning in BD during euthymic phases (Kizilkurt et al., 2018). Comorbid personality disorders are associated with a greater severity of residual thymic symptoms, even in remission (George et al., 2003), as well as with a more negative course of the illness (Fan and Hassell, 2008). Moreover, personality is modestly associated with neuropsychological performance in the general population (Bartels et al., 2012; Berggren and Derakshan, 2013; Schaie et al., 2004). However, current research does not allow us to understand the link between personality and the heterogeneity of cognitive impairment associated with BD.

The personality approach proposed by Young’s schema theory appears relevant by considering personality in relation to the person’s developmental history. People exposed to adverse experiences in early childhood can develop “Early Maladaptive Schemas” (EMS) related to these experiences (Young et al., 2003). These EMS emerge when the person’s basic affective needs are not met. The five core emotional needs are:

- secure attachments to others (safety, stability, nurturance, and acceptance);
- autonomy, competence, and sense of identity;
- freedom to express valid needs and emotions;
- spontaneity and play;
- realistic limits and self-control.

The lack of satisfaction of needs is facilitated by the interaction between contextual (childhood adversity) and biological (temperament) factors. The different patterns are built during childhood and adolescence and are enhanced throughout life. An EMS is a recurring theme or dysfunctional pattern of information processing including beliefs, emotions, and memories about oneself, others and the world (Young et al., 2003). To adapt to their EMS, individuals engage in dysfunctional behaviors. EMS impact various aspects of psychosocial functioning (Scott and Crino, 2014).

The study of EMS is limited in individuals with BD. Young’s model of Early Maladaptive Schemas (EMS) appears highly relevant in describing the experiences of individuals with bipolar disorder. According to studies, the prevalence of cumulative trauma ranges from 29% (Etain et al., 2013) to 82% (Li et al., 2014). Emotional trauma is reported in 77% of individuals with bipolar disorder (Dualibe and Osório, 2017). Furthermore, the presence of cumulative childhood trauma is a factor associated with greater severity of bipolar disorder, including an earlier age of onset, longer episode duration, a higher number of mood episodes over a lifetime, an increased likelihood of psychotic features, and a higher probability of past suicide attempts (Li et al., 2014; Rowe et al., 2024). These findings strongly support the use of Young’s schema theory, as confirmed by Dadomo et al. (2016). Theoretical frameworks suggest that early adverse experiences shape core cognitive patterns by fostering negative self-beliefs and distorted perceptions of others, which can lead to maladaptive coping mechanisms in adulthood. Such patterns may manifest as pervasive feelings of abandonment, mistrust, or worthlessness, influencing emotional regulation and behavior (Young et al., 2003).This is further validated by a recent study that demonstrated connections between specific types of trauma and the activation of particular schemas (Özdin et al., 2018). A recent literature review found that, compared to people without BD, those with BD have greater general activation of EMS, even if a specific pattern of schemas cannot be identified (Munuera et al., 2020a). A study with Danish women with bipolar 1 disorder showed a high activation of the insufficient self-control EMS (Nilsson et al., 2010). In different culture (Iran, Turkey), people with BD have higher activation for many EMS in comparison with a control group (Ak et al., 2012; Khosravi et al., 2017; Özdin et al., 2018; Korkmaz et al., 2024). But, differences are not observed on the same EMS. Such different results might be related to culture but also with study methodology. Most studies compared a group of people with BD to a control group, or to a group of people with unipolar disorder. Previous studies do not consider clinical heterogeneity by examining people with BD as a homogeneous group. It seems relevant to define homogeneous subgroups of individuals according to EMS types and explore if they differ on clinical or functional criteria. This is consistent with a personalized clinical approach (Snyderman and Yoediono, 2006). A previous study showed that the activation of specific EMS clarifies the singularity of each remission profile in individuals with BD (Munuera et al., 2020b). However to date, no study has proposed such a centered approach of EMS profiles related to a global description of clinical state during euthymic phase, including symptomatology, psychosocial functioning, and neuropsychological functioning. To the best of our knowledge, only one study has reported that the level of cognitive complaints in older adults with subjective cognitive decline was significantly associated with EMS in the “Impaired autonomy, competence, and sense of identity” domain, with a lack of significant correlation between EMS activation and objective performance in episodic memory (Tandetnik et al., 2017). Relationships between EMS and neuropsychological performances remain to be explored in BD.

Several theoretical arguments suggest a link between EMS and cognitive and social functioning, for example because the impact of compensatory behaviors on social functioning, or because issues with cognitive and social functioning make people more prone to activation of EMS. The main objective of this study was to identify homogeneous profiles of people with BD in euthymic phase with respect to EMS activation, and to compare the different subgroups (clusters) with residual symptoms, psychosocial functioning, and neuropsychological performance. We hypothesize that more residual depressive symptoms and more psychosocial and cognitive functioning impairment would be found in subtypes with the highest activation of EMS. This study has several implications for clinical practice. In particular, if the results support the hypotheses, they could improve therapeutic recommendations. For example, one perspective is to highlight the relevance of referring patients to functional remediation for cognitive impairment (Bonnin et al., 2016) and/or schema therapy (Dadomo et al., 2016; Ociskova et al., 2022). These different interventions have shown relevant results, and the aim now is to establish them as tools to enhance personalized psychiatry.

## 2 Materials and methods

### 2.1 Study design and recruitment

This was a transversal study including patients recruited from the FACE-BD (FondaMental Advanced Centers of Expertise for Bipolar Disorders) cohort within the BD Expert Center of Versailles. The BD Expert Centers were set up by the Fondation FondaMental,^1^ funded by the French Ministry of Research and the French Ministry of Health to build an infrastructure and provide resources to follow clinical cohorts. This cohort has been extensively described in a previous paper (Henry et al., 2015).

For this study, all procedures complied with the ethical standards of the national and institutional committees on human experimentation and the 1975 Declaration of Helsinki (Article 20), revised in 2008. The study was approved by the local ethics committee (Comité de Protection des Personnes Île de France IX) on January 18, 2010, under French laws on non-interventional studies (observational studies without risk, constraint, or additional or unusual procedures regarding diagnosis, treatment or follow-up). All patients were given an informational letter but waived the requirement for written informed consent. Verbal consent was witnessed and formally recorded. Regarding the procedure, all measrures were performed during the assessment at the expert center.

### 2.2 Participants

Inclusion criteria were to be between 18 and 65 years old and to be outpatients with bipolar I or bipolar II disorder or not otherwise specified bipolar disorder (NOS). BD was diagnosed via the Structured Clinical Interview for DSM-IV-TR Axis I Disorders (SCID-I/P; First, 2002) by trained professional clinicians. Exclusion criteria were a history of neurological or sensory disorders, dyslexia, dysorthographia, dyscalculia, dysphasia, dyspraxia, language delay, substance use disorders in the previous month, and electroconvulsive therapy in the previous year. Non-inclusion criteria were to be in acute depressive or manic episode according to the DSM-IV-TR criteria (APA, 2000). Euthymic phase at the time of testing was also confirmed according to the DSM-IV-TR criteria (APA, 2000), with a cut-off score of 10 on both the Montgomery-Asberg Depression Rating Scale (MADRS; Montgomery and Åsberg, 1979) and the Young Mania Rating Scale (YMRS; Young et al., 1978).

### 2.3 Measurement

#### 2.3.1 Sociodemographic data and clinical assessment

Four socio-demographic characteristics were collected: age, gender, marital status, and education level (measured in years).

To characterize disorder severity, we collected the age at onset, the number of previous episodes (mixed, hypomanic, manic, and depressive), the subtype of BD (type I or non-type I including type II and not otherwise specified), history of psychotic symptoms (present/absent) and history of substance use disorder (present/absent). We also specified the predominant mood polarity (3 levels: manic, depressive or indeterminate valence), the presence or absence of rapid cycling, the presence or absence of complete remission between episodes, and the time elapsed since the end of the last characterized mood episode (more/<3months).

The severity of the current clinical condition was assessed by the Clinical Global Impression scale (CGI) severity score (Busner and Targum, 2007), on a scale from 1 (“normal”) to 7 (“among the most ill patients”). We used a yes/no format to record whether the patient was taking lithium, mood stabilizer anticonvulsants, antipsychotics, antidepressants or anxiolytics at the time of assessment. The presence of childhood trauma was assessed with the Childhood Trauma Questionnaire (CTQ) (Paquette et al., 2004).

Symptomatology at the time of assessment was measured by the MADRS depression (Montgomery and Åsberg, 1979) score and the YMRS mania score (Young Mania Rating Scale, Young et al., 1978). The state of anxiety at the time of the assessment was measured with the State–Trait Anxiety Inventory (STAI) (Julian, 2011).

#### 2.3.2 Early maladaptive schemas (EMS)

The Young Schema Questionnaire-Short Form 3 (YSQ-S3) was used to assess EMS. This self-report questionnaire includes 90 items rated on a 6-point scale ranging from 1 (“completely untrue of me”) to 6 (“describes me perfectly”) (Young, 2005).

There are 18 EMS assessed, divided into 5 domains:

1. Disconnection and Rejection (abandonment; mistrust/abuse; emotional deprivation; defectiveness/shame; social isolation)
2. Impaired Autonomy and Achievement (dependency/incompetency; vulnerability to harm/illness; enmeshment/undeveloped self; failure)
3. Impaired Limits (entitlement/grandiosity; lack of self-control/self-discipline)
4. Other-directedness (subjugation; self-sacrifice; approval/recognition-seeking)
5. Hypervigilance and Inhibition (negativity/pessimism; emotional inhibition; unrelenting standards; punitiveness)

This tool has demonstrated adequate validity and reliability in an adult sample with and without mental illness (Bouvard et al., 2018). The average individual scores on each schema were transformed into standardized scores relative to the norm (Bouvard et al., 2018).

#### 2.3.3 Functional outcomes

Psychosocial functioning was assessed by the Functioning Assessment Short Test (FAST; Rosa et al., 2007), an interviewer-administered instrument. It assesses the functional impairment of patients in six functioning areas through 24 items: autonomy, occupational functioning, cognitive functioning, financial issues, interpersonal relationships, and leisure time. Each item is rated on a 0–3 scale (0 = no difficulty; 3 = severe difficulty). The higher the score, the higher the psychosocial impairment. Participants were also evaluated on the Global Assessment of Functioning scale (GAF), scored from 0 (high global impaired functioning) to 100 (good global functioning) (Jones et al., 1995).

Finally, health-related quality of life was assessed with the EuroQol-5 Dimension (EQ-5D-3L). The EQ-5D-3L is a preference-based measure developed to describe and evaluate health across a wide range of disease areas (EuroQol Group, 1990). It is based on one question for each of the five dimensions including mobility, self-care, usual activities, pain/discomfort, and anxiety/depression (Balestroni and Bertolotti, 2012). Each dimension has three levels: no problems, some problems, and extreme problems. EQ-5D health states were converted into a single summary number, the index value obtained with the time trade-off évaluation technique (Chevalier and de Pouvourville, 2013). It reflects how good or bad a health state is according to the preferences of the general population of a country/region.

#### 2.3.4 Neuropsychological assessment

Neuropsychologists administered the tests in a systematic order. The tests lasted a total of 120 min, including breaks of 5–10 min. The test battery selected was in accordance with the recommendations of the International Society for Bipolar Disorders (ISBD; Yatham et al., 2010), consisting of 11 tests that assess six cognitive domains: verbal memory, working memory, executive functions, processing speed, attention, and reasoning. Verbal memory was assessed by the California Verbal Learning Test (CVLT) (Woods et al., 2006). Working memory was assessed by the Digit Memory subtest of the WAIS-III (Wechsler, 1997a) and the Visual–Spatial span of the WMS-III (Wechsler Memory Scale - 3rd edition; Wechsler, 2001). Executive functions were assessed by the TMT-B (Trail Making Test - Part B), the Stroop Word and Color Test, and the Verbal Fluency Test (Reitan, 1958; Lezak et al., 2004). Processing speed was assessed by the WAIS-III Codes and Symbols subtests, the Stroop test, and the TMT-A (Trail Making Test - Part A). Attention was assessed by the CPT-II (Conners’ Continuous Performance Test - 2nd edition; Conners, 2000). Finally, reasoning was assessed by the Vocabulary and Matrices subtests of the WAIS-III (Wechsler, 1997b). Raw scores were transformed into normatively corrected standardized z-scores. Higher scores reflect better performance. We calculated a mean score for each of the six cognitive domains, as the average of the z-scores for each measure within a domain.

### 2.4 Statistical analysis

R software was used to perform statistical analyses (R Core Team, 2019). A hierarchical ascendant cluster analysis was conducted to identify homogeneous groups of people with BD based on the activation of the 18 EMS. We used Ward’s minimum variance as a linking criterion. The optimal number of clusters was determined by visual inspection of the dendrogram, the D-index method (Lebart et al., 2000) and Hubert’s method (Hubert and Arabie, 1985). Discriminant analysis was performed to test the validity of the clusters (Wilks’ lambda test for canonical correlations, with Rao approximation) (Mardia et al., 1979).

Multivariate analysis of variance (MANOVA) with a Wilks test was used to test for differences in EMS activation between clusters. *Post-hoc* pairwise comparisons were performed when necessary to identify differences between two clusters for a specific EMS, with a correction for multiple comparisons using the false discovery rate procedure (Benjamini and Hochberg, 1995).

To determine whether clusters differed for sociodemographic, clinical, neuropsychological, and functional variables, we performed a succession of *χ*^2^ tests and ANOVAs. Pairwise *post-hoc* comparisons were performed when necessary to identify differences between two clusters for each variable, using again the false discovery rate procedure for multiple comparison correction.

Finally, to determine whether the effect of clusters on our variables of interest (residual depressive symptoms, psychosocial functioning, and neuropsychological performance) remained statistically significant after controlling for all covariates, we performed several multiple regressions. First, missing data were estimated using multiple imputations (50 imputations) by Markoff chain equations with the MICE function (Van Buuren and Groothuis-Oudshoorn, 2011). Next, we selected as dependent variables the cognitive and functioning measures that were significantly associated with EMS cluster type in the bivariable analyses described above. Then we selected as covariates of interest those that were associated with cluster type with a *p* < 0.2 in the bivariable analyses described above.

## 3 Results

### 3.1 Participants

This study included 111 participants. Overall, 55.9% of the sample was female. The mean age was 39.6 (±11.6), 56% of the sample had a marital partner; 39.6% suffered from bipolar II disorder or NOS and 60.4% suffered from bipolar I disorder (see Table 1).

**Table 1:** Sociodemographic and clinical characteristics of the sample.

	Variable	Mean (SD)/Percentage
Sociodemographic data	Age (years)	39.6 (11.6)
	Gender (male)	44.1%
	Educational level (years)	14.8 (2.3)
	Marital status	56%
Clinical characteristics of bipolar disorder	BP1	39.6%
	Total number of thymic episodes	8.4 (8.8)
	Predominant manic valence	16.7%
	Predominant depressive valence	36.9%
	Undetermined valence	47.4%
	Age of onset (years)	22.8 (7.7)
	Psychotic features	24.7%
	Rapid cycling	7.7%
	Complete remission between episodes	74.8%
	CGI	4.5 (0.7)
	MADRS	4.2 (3.2)
	YMRS	2.1 (2.8)
	End of last episode >3 months	76.1%
Treatments	Antidepressant	21.6%
	Anticonvulsant thymoregulators	26.1%
	Lithium	14.4%
	Antipsychotic	16.2%
	Anxiolytic	16.2%
Psychosocial markers of functioning	FAST (psychosocial functioning)	14.5 (10.6)
	GAF	64.6 (12.1)
	Work	71.7%
	Independent housing	75.2%
	EQ-5D	0.8 (0.2)
Other clinical characteristics	CTQ	41.8 (13.7)
	STAI-YA	38 (13.1)
	Substance use disorder (lifetime)	17.8%

SD, standard deviation; BP1, Bipolar Disorder type 1; CGI, Clinical Global Impression scale; MADRS, Montgomery-Åsberg Depression Rating Scale; YMRS, Young Mania Rating Scale; FAST, Functioning Assessment Short Test; GAF, Global Assessement Functioning; EQ-5D, EuroQol-5D: quality of life; CTQ, Childhood Trauma Questionnaire: childhood trauma score; STAI forme YA, State Anxiety Inventory forme YA.

The average age of onset of the disorder was 22.8 years. Participants reported an average of 8.4 episodes, and nearly 1 in 4 patients (24.7%) had psychotic symptoms during episodes (Table 1).

The average STAI-YA was 38 (±13.1), corresponding to a moderate level of anxiety.

### 3.2 Early maladaptive schemas (EMS)

Cronbach’s *α* internal consistencies for each schema are reported in Table 2. The sample was characterized by an average hyperactivation of EMS (0.30 SD ± 0.27). The most hyperactivated EMS were emotional deprivation (0.80 SD ± 1.39) and defectiveness/shame (0.75 SD ± 1.57). The lowest activations were found for the EMS mistrust/abuse (−0.04 SD ± 1.11), failure (0.05 SD ± 1.1), vulnerability to harm/disease (−0.16 SD ± 1.04) and emotional inhibition (0.01 SD ± 0.95).

**Table 2:** Internal consistency metrics (Cronbach’s α) for the YSQ-S3 subscales.

EMS	Alpha de Cronbach	CI lower	CI upper
Emotional deprivation	0.74	0.65	0.81
Abandonment/Instability	0.82	0.76	0.87
Mistrust/Abuse	0.81	0.75	0.86
Social isolation/Alienation	0.84	0.79	0.89
Defectiveness/Shame	0.88	0.84	0.91
Failure	0.85	0.8	0.89
Dependence/Incompetence	0.74	0.65	0.81
Vulnerability to harm or illness	0.72	0.62	0.79
Enmeshment/undeveloped self	0.58	0.44	0.69
Entitlement/Grandiosity	0.62	0.49	0.72
Insufficient self-control/Self-discipline	0.73	0.64	0.8
Subjugation	0.79	0.72	0.85
Self-sacrifice	0.76	0.69	0.83
Approval-seeking/Recognition-seeking	0.69	0.59	0.77
Emotional inhibition	0.79	0.72	0.84
Unrelenting standards/Hypercriticalness	0.51	0.34	0.64
Negativity/Pessimism	0.78	0.71	0.84
Punitiveness	0.69	0.59	0.77

YSQ, Young Schemas Questionnaire; EMS, Early Maladaptive Schemas; CI, confidence interval.

### 3.3 Functional outcomes

The mean global disease severity score for the sample was 4.5 (± 0.7) (between “moderately ill” and “obviously ill”). The mean GAF functioning score was 64.6 (± 12.1) (some mild symptoms). The mean FAST score (psychosocial functioning) was 14.5 (± 10.6). The mean quality of life score was 0.82 (± 0.18), which was below scores reported in the general French population (ranging from 0.95 in the 18–24 years old class to 0.85 in the 55–64 years old class; Janssen et al., 2019).

### 3.4 Neuropsychological assessment

Lower cognitive performance was found for attention (−0.45 SD) and higher for reasoning (0.68 SD) and verbal memory (0.59 SD). Working memory (−0.02 SD) and processing speed (0.07 SD) were close to the norm (see Table 3).

**Table 3:** Standardized mean neuropsychological performance scores of the sample.

Function	Test	Variable (Test)	Mean (SD)
Verbal memory			0.59 (0.81)
	CVLT	Immediate recall	0.82 (1.19)
		Short delay free recall	0.49 (1.08)
		Long delay free recall	0.50 (1.05)
		Total recognition	0.54 (0.39)
Working memory			−0.02 (0.68)
	Digit span – WAIS-III	Forward and backward	−0.06 (0.89)
	Spatial span – WMS-III	Forward	−0.01 (0.95)
		Backward	0.02 (0.79)
Executive functions			0.01 (0.79)
	Trail making test	Part B	0.12 (1.22)
	Stroop test	Word and color	0.25 (0.99)
	Verbal fluency	Lexical	0.02 (1.01)
		Catégoriel	−0.33 (0.98)
Processing speed			0.07 (0.66)
	WAIS-III	Coding	0.00 (1.01)
		Symbols	0.00 (0.94)
	Test de Stroop	Color	0.00 (0.73)
		Word	0.00 (0.81)
	TMT	Part A	0.00 (0.69)
Attention			−0.45 (0.63)
	CPT-II	Omissions	−0.91 (1.14)
		Commissions	−0.38 (1.12)
		Variability	−0.13 (1.05)
		Detectability	−0.36 (1.00)
Reasoning			0.68 (0.70)
	WAIS-III	Vocabulary	0.96 (0.88)
		Matrix reasoning	0.50 (0.79)

SD, Standard deviation; CVLT, California verbal learning test; WMS, Wechsler memory scale; TMT, Trail making test; CPT-II, Conners’ continuous performance test II.

### 3.5 Results of cluster analysis

**Figure 1:**
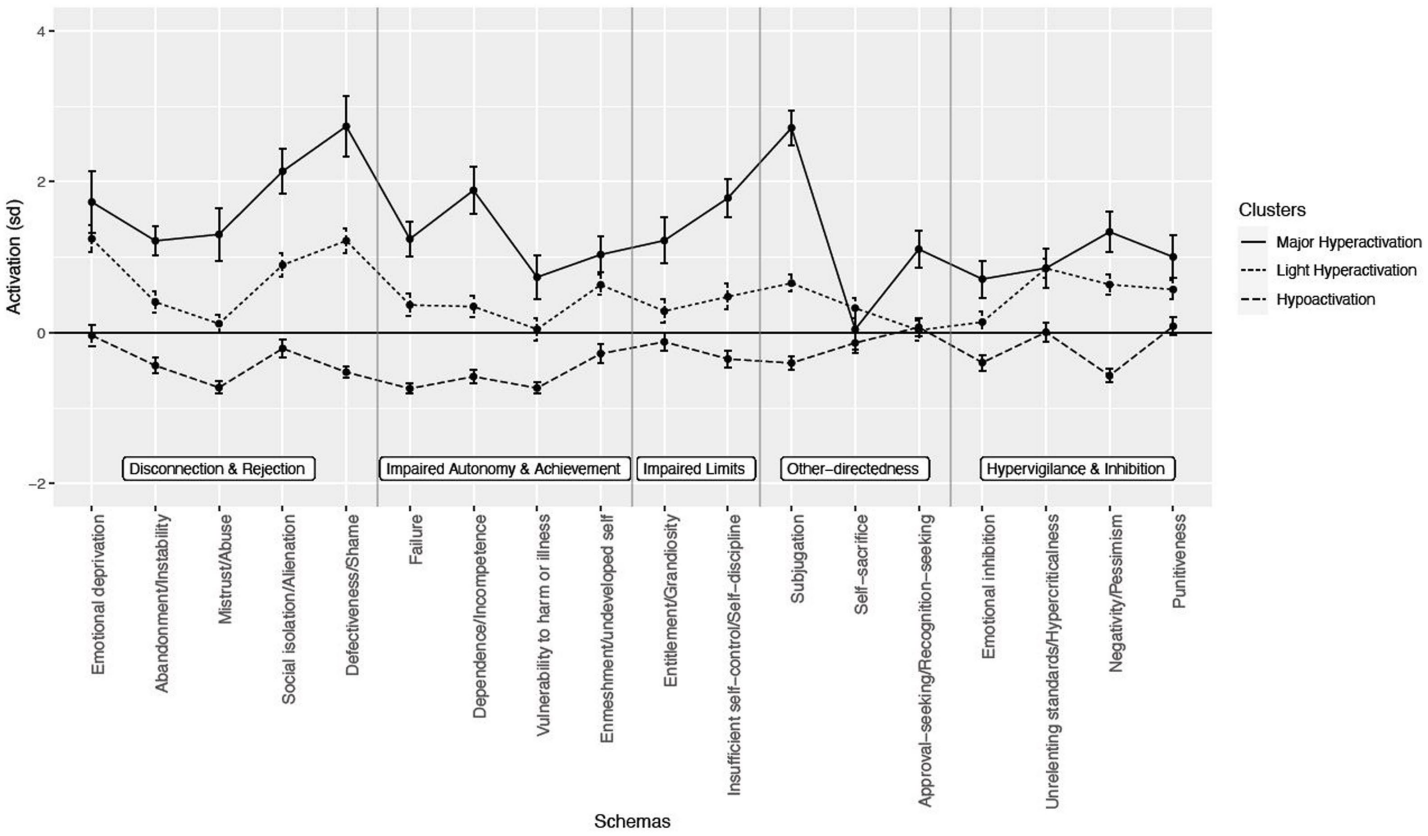
Profiles of Early Maladaptive Schemas in bipolar euthymics patient.

Using cluster analysis, 3 profiles were identified. The discriminant analysis revealed the presence of two discriminant factors, explaining, respectively, 91.4 and 8.6% of the discriminant power in cluster membership (trace proportion). For the first function, Wilks’ *λ* was 0.10 [*F*(36, 182) = 10.8, *p* < 0.001] and for the second function, Wilks’ λ was 0.66 [*F*(17, 92) = 7.5, *p* < 0.001]. A total of 96.4% of participants were correctly classified according to these two discriminant functions. The two EMS most strongly correlating with the first discriminant function were subjugation (Pearson correlation coefficient *r* = 0.87) and defectiveness/shame (*r* = 0.81). The EMS most strongly correlating with the second discriminant function was approval/recognition-seeking (*r* = 0.45) (see Figure 1).

Mean EMS activations per cluster and statistics for pairwise comparisons are summarized in Tables 4, 5. The first cluster was composed of 40.5% of the sample (n = 45). In this cluster, 12 out of 18 EMS were hypoactivated. This cluster was named *Hypoactivation*.

**Table 4:** Main effects of EMS cluster membership on sociodemographic and clinical variables.

Variable	Statistic	*p*
Age	*F*(2, 108) = 2.4	0.098
Gender (percentage of male)	X^2^(2) = 1.2	0.543
Educational level (years)	*F*(2, 107) = 0.5	0.623
Bipolar 1 disorder	X^2^(2) = 5.9	0.051
Number of episodes	*F*(2, 75) = 0.4	0.704
Manic episodes predominant	X^2^(4) = 7.7	0.102
Age of onset	*F*(2, 104) = 3.1	0.051
Psychotic symptoms	X^2^(2) = 1.4	0.485
Rapid cycling	X^2^(2) = 1.2	0.542
Full remission between episodes	X^2^(2) = 3	0.226
CGI	*F*(2, 108) = 3.1	0.048*
MADRS	*F*(2, 108) = 2.4	0.095
YMRS	*F*(2, 108) = 0.6	0.555
End of last episode >3 months	X^2^(2) = 7.8	0.021*
Antidepressant	X^2^(2) = 0.1	0.939
Anticonvulsant	X^2^(2) = 0.9	0.646
Lithium	X^2^(2) = 4	0.135
Antipsychotic	X^2^(2) = 3.8	0.15
Anxiolytic	X^2^(2) = 1.7	0.428
CTQ (trauma)	*F*(2, 108) = 6.8	0.002**
STAI form YA (anxiety)	*F*(2, 108) = 23.8	<0.001***
Substance use disorder (lifetime)	X^2^(2) = 7.9	0.019*

EMS, Early Maladaptive Schemas; CGI, Clinical Global Impression scale: Severity score; MADRS, Montgomery-Åsberg Depression Rating Scale; YMRS, Young Mania Rating Scale; CTQ, Childhood Trauma Questionnaire; STAI form YA, State Anxiety Inventory form YA; *p < 0.05; **p < 0.01; ***p < 0.001.

**Table 5:** Mean EMS activations per cluster and statistics for pairwise comparisons.

EMS	Hypo-activation	Light activation	Major hyper-activation	Hypo-activation vs. light activation	Hypo-activation vs. major hyper-activation	Light activation vs. major hyper-activation
	**Mean (SD)**	**Mean (SD)**	**Mean (SD)**	***t*(df)** ***p***	***t*(df)** ***p***	***t*(df)** ***p***
Emotional deprivation	−0.04 (0.95)	1.24 (1.23)	1.73 (1.66)	*t*(92) = −5.6 <0.001	*t*(60) = −5.2 <0.001	*t*(64) = −1.3 0.154
Abandonment/Instability	−0.44 (0.7)	0.41 (1)	1.22 (0.79)	*t*(92) = −4.7 <0.001	*t*(60) = −8 <0.001	*t*(64) = −3 0.001
Mistrust/Abuse	−0.73 (0.54)	0.12 (0.86)	1.3 (1.44)	*t*(92) = −5.6 <0.001	*t*(60) = −8.1 <0.001	*t*(64) = −4.1 <0.001
Social isolation/Alienation	−0.21 (0.84)	0.9 (1.13)	2.14 (1.23)	*t*(92) = −5.4 <0.001	*t*(60) = −8.6 <0.001	*t*(64) = −3.8 <0.001
Defectiveness/Shame	−0.52 (0.49)	1.22 (1.17)	2.73 (1.66)	*t*(92) = −9.3 <0.001	*t*(60) = −12 <0.001	*t*(64) = −4.1 <0.001
Failure	−0.74 (0.43)	0.37 (1.04)	1.24 (0.97)	*t*(92) = −6.6 <0.001	*t*(60) = −11.2 <0.001	*t*(64) = −3 <0.001
Dependence/Incompetence	−0.58 (0.64)	0.35 (1.02)	1.89 (1.27)	*t*(92) = −5.2 <0.001	*t*(60) = −10.1 <0.001	*t*(64) = −5 <0.001
Vulnerability to harm or illness	−0.73 (0.51)	0.05 (1.06)	0.74 (1.17)	*t*(92) = −4.5 <0.001	*t*(60) = −6.9 <0.001	*t*(64) = −2.2 0.008
Enmeshment/undeveloped self	−0.28 (0.82)	0.64 (0.95)	1.04 (0.99)	*t*(92) = −5 <0.001	*t*(60) = −5.3 0 < 0.001	*t*(64) = −1.5 0.12
Entitlement/Grandiosity	−0.12 (0.83)	0.29 (1.06)	1.22 (1.25)	*t*(92) = −2.1 0.053	*t*(60) = −4.9 <0.001	*t*(64) = −3 0.002
Insufficient self-control/Self-discipline	−0.35 (0.76)	0.48 (1.19)	1.78 (1.06)	*t*(92) = −4 <0.001	*t*(60) = −8.8 <0.001	*t*(64) = −4 <0.001
Subjugation	−0.4 (0.64)	0.66 (0.76)	2.71 (0.97)	*t*(92) = −7.3 <0.001	*t*(60) = −14.8 <0.001	*t*(64) = −9 <0.001
Self-sacrifice	−0.13 (0.86)	0.33 (0.97)	0.04 (1.08)	*t*(92) = −2.4 0.06	*t*(60) = −0.7 0.507	*t*(64) = 1 0.438
Approval-seeking/Recognition-seeking	0.07 (0.81)	0.03 (0.93)	1.11 (1.01)	*t*(92) = 0.2 0.829	*t*(60) = −4.2 <0.001	*t*(64) = −4 <0.001
Emotional inhibition	−0.4 (0.7)	0.14 (0.97)	0.71 (1.02)	*t*(92) = −3.1 0.006	*t*(60) = −4.9 <0.001	*t*(64) = −2.1 0.023
Unrelenting standards/Hypercriticalness	0.01 (0.89)	0.85 (0.87)	0.86 (1.08)	*t*(92) = −4.7 <0.001	*t*(60) = −3.2 0.002	*t*(64) = 0 0.991
Negativity/Pessimism	−0.57 (0.62)	0.64 (0.93)	1.34 (1.09)	*t*(92) = −7.3 <0.001	*t*(60) = −8.7 <0.001	*t*(64) = −2.5 0.004
Punitiveness	0.09 (0.8)	0.57 (0.9)	1 (1.17)	*t*(92) = −2.8 0.015	*t*(60) = −3.5 0.002	*t*(64) = −1.6 0.093

SD, standard deviation; t, value for each comparison; df, Degrees of freedom associated with each comparison; *p*, *p*-value associated with each comparison.

The second cluster was composed of 44.1% of the sample (n = 49). Overall, EMS were slightly more activated compared to the norm, especially entitlement/grandiosity, social isolation, and emotional deprivation. On the other hand, the EMS mistrust/abuse, vulnerability to harm/illness, approval/recognition-seeking, and emotional inhibition were in the activation norm. This cluster was labeled *Light activation*.

The third cluster was composed of 15.3% of the sample (*n* = 17). Almost all of the EMS were markedly hyperactivated except for the self-sacrifice EMS. The subjugation, defectiveness/shame, and social isolation EMS were particularly hyperactivated. This cluster was named *Major hyperactivation*.

### 3.6 Effect of clusters

#### 3.6.1 Effect of cluster membership on clinical variables

There was a statistically significant effect of cluster membership on the following clinical variables: disorder severity [*F*(2, 108) = 3.1, *p* = 0.048]; at least 3 months’ time elapsed from the last episode [X^2^(2) = 7.8, *p* = 0.021]; childhood trauma score [*F*(2, 108) = 6.8, *p* = 0.002]; state anxiety [*F*(2, 108) = 23.8, *p* < 0.001]; and lifetime history of substance use disorders [X^2^(2) = 7.9, *p* = 0.019]. In contrast, residual thymic symptoms were not significant for either depressive symptoms [*F*(2, 108) = 2.4, *p* = 0.095] or manic symptoms [*F*(2, 108) = 0.6, *p* = 0.555] (see Table 6).

**Table 6:** *Post-hoc* pairwise *t*-tests of EMS activation clusters on clinical variables.

Clinical variables	Hypo-activation	Light activation	Major hyper-activation	Hypo-activation vs. light activation	Hypo-activation vs. major hyper-activation	Light activation vs major hyper-activation
	Mean (SD) %	Mean (SD) %	Mean (SD) %	*t* or *χ*^2^ (df) *p* Cohen’s d or Pearson’s ϕ	*t* or *χ*^2^ (df) *p* Cohen’s d or Pearson’s ϕ	*t* or *χ*^2^ (df) *p* Cohen’s d or Pearson’s ϕ
CGI	4.47 (0.87)	4.45 (0.68)	4.94 (0.43)	*t*(92) = 0.1 0.907 0.02	*t*(60) = −2.1 0.038 −0.61	*t*(64) = −2.8 0.038 −0.79
End of last episode >3 months	84.4	77.1	50	*χ*^2^(1) = 0.4 0.525 0	*χ*^2^(1) = 5.8 0.048 0.33	*χ*^2^(1) = 3 0.123 0.22
CTQ (trauma)	36.67 (9.72)	44 (14.45)	49 (15.99)	*t*(92) = −2.9 0.011 −0.59	*t*(60) = −3.7 0.004 −1.05	*t*(64) = −1.2 0.175 −0.34
STAI form YA (anxiety)	29.64 (7.88)	42.04 (12.34)	48.41 (13.96)	*t*(92) = −5.7 <0.001–1.19	*t*(60) = −6.7 <0.001–1.9	*t*(64) = −1.8 0.043 −0.5
Substance use disorder (lifetime)	11.1	15.6	41.2	*χ*^2^(1) = 0.1 0.756 0	*χ*^2^(1) = 5.3 0.062 0.31	*χ*^2^(1) = 3.3 0.105 0.24

EMS, Early Maladaptive Schemas; CGI, Clinical Global Impression scale; CTQ, Childhood Trauma Questionnaire; STAI form YA, State Anxiety Inventory form YA; SD, standard deviation; t, value for each comparison; df, degrees of freedom associated with each comparison; *p*, *p*-value associated with each comparison; *χ*^2^(Chi^2^).

*Post-hoc* pairwise *t*-tests of EMS activation clusters on clinical variables are reported in Table 6. The Major hyperactivation cluster had a significantly higher disorder severity score than the Hypoactivation [*t*(60) = 2.1, corrected *p* = 0.038], and *Light activation* clusters [*t*(64) = 2.8, corrected *p* = 0.038]. The *Hypoactivation* cluster had a lower childhood trauma score than the *Light activation* [*t*(92) = −2.9, corrected *p* = 0.011] and *Major hyperactivation* [*t*(60) = −3.7, corrected *p* = 0.004] clusters. The *Light activation* cluster also differed from the *Hypoactivation* cluster by a lesser proportion of participants having more than 3 months’ time elapsed since last episode [X^2^(1) = 5.8, *p* = 0.048] and a higher intensity of anxiety symptomatology [*t*(60) = 6.7, corrected *p* < 0.001]. Furthermore, the anxiety symptom intensity was significantly different for all *post-hoc* contrasts. Participants in the *Major hyperactivation* cluster had higher anxiety scores than those in the other clusters [respectively compared to *Hypoactivation*: *t*(60) = −6.7, corrected *p* < 0.001; and *Light activation*: *t*(64) = −1.8, corrected *p* = 0.043]. Individuals in the *Light activation* cluster had higher anxiety scores than those in the *Hypoactivation* cluster [*t*(92) = −5.7, corrected *p* < 0.001]. Finally, the *Major hyperactivation* cluster had a marginally higher lifetime history of substance use disorders than the Hypoactivation cluster [X^2^(1) = 5.3, *p* = 0.062].

#### 3.6.2 Effect of cluster membership on sociodemographic variables and psychosocial functioning

There was a statistically significant effect of the clusters on the following sociodemographic and psychosocial functioning variables (see Table 7): marital status [X^2^(2) = 8.4, *p* = 0.015], FAST [*F*(2, 107) = 11.7, *p* < 0.001] and GAF [*F*(2, 108) = 9, *p* < 0.001] functioning scores, and quality of life [*F*(2, 89) = 12.9, *p* < 0.001]. Participants in the *Hypoactivation* cluster were significantly more likely to be in a relationship [X^2^(1) = 6.3, corrected *p* = 0.037], and were characterized by a better psychosocial functioning measured with the FAST [*t*(91) = −3.6, corrected *p* = 0.001] and a better quality of life [*t*(78) = 4.3, corrected *p* < 0.001] than participants in the *Light activation* cluster. The *Major hyperactivation* cluster also presented significantly worse functioning scores assessed with the FAST [*t*(59) = −5, corrected *p* < 0.001] and the GAF [*t*(60) = −4.3, corrected *p* < 0.001], and a worse quality of life score [*t*(51) = −5.1, corrected *p* < 0.001] than the *Hypoactivation* cluster. Only the GAF score was lower in the *Major hyperactivation* cluster than in the *Light activation* cluster [*t*(64) = −3.0, corrected *p* = 0.006] (see Table 8).

**Table 7:** Main effects of EMS cluster membership on functioning variables.

Variable	Statistic	*p*
Marital status	X^2^(2) = 8.4	0.015*
Work	X^2^(2) = 0.4	0.815
Independent housing	X^2^(2) = 0.2	0.918
Psychosocial functioning FAST	*F*(2, 107) = 11.7	<0.001***
Global functioning GAF	*F*(2, 108) = 9	<0.001***
Quality of life (EQ-5D)	*F*(2, 89) = 12.9	<0.001***

EMS, Early Maladaptive Schemas; FAST, Functioning Assessment Short Test; GAF, Global Assessment of Functioning: functioning scales; EQ-5D, EuroQol-5D: quality of life; *p < 0.05; **p < 0.01; ***p < 0.001.

**Table 8:** *Post-hoc* pairwise *t*-tests of EMS activation clusters on functioning variables.

Functioning variables	Hypo-activation	Light activation	Major Hyper-activation	Hypo-activation vs. light activation	Hypo-activation vs. major hyper-activation	Light activation vs major hyper-activation
	Mean (SD) %	Mean (SD) %	Mean (SD) %	*t* or *χ*^2^ (df) *p* Cohen’s d or Pearson’s ϕ	t or *χ*^2^ (df) *p* Cohen’s d or Pearson’s *ϕ*	*t* or *χ*^2^ (df) *p* Cohen’s d or Pearson’s ϕ
Marital status	72.7	44.9	43.8	*χ*^2^ (1) = 6.3 0.037 0.26	*χ*^2^ (1) = 3.2 0.114 0.24	*χ*^2^ (1) = 0 1 0
Psychosocial functioning FAST	9.48 (7.33)	16.63 (11.04)	21.65 (10.9)	*t*(91) = −3.6 0.001 −0.76	*t*(59) = −5 <0.001 −1.44	*t*(64) = −1.6 0.069 −0.46
Global functioning GAF	68.56 (11.43)	64.27 (11.43)	55 (10.25)	*t*(92) = 1.8 0.068 0.38	*t*(60) = 4.3 <0.0011.22	*t*(64) = 3 0.006 0.83
Quality of life (EQ-5D)	0.91 (0.09)	0.77 (0.19)	0.68 (0.24)	*t*(78) = 4.3 <0.0010.96	*t*(51) = 5.1 <0.0011.67	*t*(49) = 1.4 0.089 0.46

SD, standard deviation; t, value for each comparison; df, degrees of freedom associated with each comparison; *p*, *p*-value associated with each comparison; *χ*^2^ (Chi^2^).

#### 3.6.3 Effect of cluster membership on cognitive variables

Our results show no effect of cluster membership on neuropsychological performance variables (see Table 9).

**Table 9:** Main effects of EMS cluster membership on cognitive dimensions.

Variable	Statistic	*p*
Verbal memory	*F*(2, 103) = 0.1	0.937
Working memory	*F*(2, 103) = 0.3	0.757
Executive functions	*F*(2, 103) = 0.1	0.933
Processing speed	*F*(2, 103) = 0.1	0.941
Attention	*F*(2, 103) = 0.9	0.402
Reasoning	*F*(2, 103) = 0.5	0.594

EMS, Early Maladaptive Schemas.

### 3.7 Multiple regression analysis

The results of the multiple regression analyses are reported in Supplementary material. Only marital status was significantly different between the *Light activation* and *Hypoactivation* clusters [*t*(88.6) = −3.1, *p* = 0.005] and the *Major hyperactivation* and *Hypoactivation* clusters [*t*(84.5) = −3.3, *p* = 0.005], participants in the *Hypoactivation* cluster being more often in a relationship. Functioning assessed with the FAST and GAF and quality of life were not significantly associated with EMS. However, several covariates were significantly associated with the functioning scores and quality of life. Functioning measured with FAST and GAF was worse in individuals whose last episode ended <3 months ago [*t*(89.9) = −3.6, *p* = 0.001, and *t*(91) = 2.2, *p* = 0.032, respectively]. The GAF and quality of life scores were negatively associated with anxiety symptom intensity [*t*(90.9) = −2.1, *p* = 0.039, and *t*(65) = −4.5, *p* < 0.001, respectively].

## 4 Discussion

Using cluster analysis, this study examined the link between Early Maladaptive Schema (EMS) profiles, clinical characteristics, residual symptoms of bipolar disorders (BD), psychosocial functioning, and neuropsychological performance. As a main result, we found three different cluster profiles based on EMS activation. This result is in line with previous studies and sheds light on the clinical heterogeneity in BD. People with BD are not homogeneous during euthymic phases which supports the relevance of proposing personalized medicine and care. Futhermore, these different profiles were associated with clinical variables and functioning outcomes, but not with neuropsychological performance.

Firstly, a significant proportion of participants in our sample displayed functioning measures within the norm during the euthymic phase of their BD, both in terms of EMS activation and psychosocial functioning. Indeed, the *Hypoactivation* cluster, comprising 40% of the sample, was particularly characterized by hypoactivation of dependence/incompetence, failure, defectiveness/shame, mistrust/abuse, negativity/pessimism, and vulnerability to harm/illness EMS, which was associated with good levels of psychosocial functioning. Regarding the hypoactivation of EMS, people in this cluster would be less likely to be pessimistic and have negative self-perceptions than the others in our sample. Thus, they might be less prone to social stigma and self-stigma. It might explain the better functioning found in this cluster because social stigma and self-stigma have a major weight which impact the functioning of people (Latalova et al., 2013; Au et al., 2019). Lastly, the activation of the self-sacrifice, entitlement/grandiosity, enmeshment/undeveloped self, emotional deprivation, punitiveness, approval/recognition-seeking EMS remains very close to the norm and lower than the other participants in our sample.

A second cluster, *Light activation*, composed 44.1% of the sample. Overall, people in this cluster were more activated on the different EMS than the norm, especially for entitlement/grandiosity, social isolation, and emotional deprivation. The latter two EMS belonging to the Separation and Rejection domain, people with activated EMS in this domain are more likely to have difficulty forming secure and satisfying relationships with others (Young et al., 2003). They feel that their needs for stability, security, attention, love and belonging will never be met. They highly tend to have difficulties in relationships with others, even in therapeutic relationships. Assessing and working on these EMS could help for increase therapeutic alliance.

The last identified cluster, *Major hyperactivation* composed a minority of participants (15.3%). Except for the self-sacrifice EMS, all the other EMS were markedly hyperactivated in this cluster, especially subjugation, defectiveness/shame, and social isolation. The activation of the defectiveness/shame (Özdin et al., 2018; Khosravi et al., 2017; Richa and Richa, 2013; Hawke and Provencher, 2012) and emotional deprivation (Khosravi et al., 2017; Richa and Richa, 2013; Hawke and Provencher, 2012) EMS is inconsistent across studies, but the social isolation EMS is one of the most frequently hyperactivated in people with BD (Ak et al., 2012; Özdin et al., 2018; Khosravi et al., 2017; Richa and Richa, 2013; Hawke and Provencher, 2012). The activation of this EMS suggests that people may experience difficulties in establishing or maintaining satisfactory social relationships. The subjugation EMS corresponds to excessive submission to the control of others, because the subject feels forced to do so, usually to avoid anger, retaliation, or abandonment. There is repressed anger causing symptoms such as passive/aggressive behavior or uncontrolled outbursts of anger. Indeed, it is well known that BD are associated with emotion regulation disturbances (De Prisco et al., 2022; Dodd et al., 2019; Kurtz et al., 2021; Miola et al., 2022). The lifetime history of substance use disorders was higher in the *Major hyperactivation* cluster, highlighting the critical issue of dual diagnosis in BD. This result is supported by a recent study reporting EMS to be overactivated in individuals with alcohol use disorders (Rubio-Escobar et al., 2024).

People with hypoactivated EMS seem to have less difficulties in their daily life than people with hyperactivated EMS. Indeed, we found that psychosocial functioning, quality of life, and marital life were generally better in the *Hypoactivation* cluster than in the other clusters. These results are consistent with a recent review suggesting that schema therapy was effective in improving the quality of life for personal disorders (Zhang et al., 2023). Moreover, clinical characteristics were less severe in this cluster than the others clusters (e.g., less anxious, less severe bipolar illness, last thymic episode further away).

However, only marital status remained significantly associated with cluster memberships in the multiple regression analysis: people with hyperactivated EMS seem to have more difficulty being and remaining in a marital relationship than people with hypoactivated EMS. The hyperactivation of EMS in the Separation and Rejection domain in the *Light activation* and *Major hyperactivation* clusters could explain the difficulty in establishing and maintaining a marital relationship, in line with the meta-analysis conducted by Janovsky et al. (2020) and the study by Dupouy et al. (2023) that highlights the contribution of the Separation and Rejection domain in relationship dysfunction. We know that a significant proportion of people with BD do not regain full functioning, particularly in the social domain, after an affective episode (MacQueen et al., 2001). This study emphasizes that marital life is a relevant functioning domain to target in clinical practice and to understand in relation to patients’ EMS. Based on these results, it therefore seems legitimate to recommend an EMS assessment for individuals with BD disorder in the euthymic phase who report difficulties in establishing a stable marital relationship, and subsequently, recommend schema therapy (Young et al., 2003).

Overall, this study showed that half of people with BD have activated EMS. According to Young’s schema theory (Young et al., 2003), EMS emerge from childhood trauma. Thus, the high prevalence of trauma in this population (Etain et al., 2010; Garno et al., 2005; Janiri et al., 2015) may be a cause of fragility in their construction throughout their lives, and thus favor the development of EMS. This hypothesis is supported by the results of this study showing that the clusters differed on the presence of childhood trauma in bivariable analysis. Particularly, the *Hypoactivation* cluster showed a lower childhood trauma score than the two other clusters. This result is consistent with scientific literature showing an association between childhood maltreatment and EMS (Carr and Francis, 2010). Furthermore, because there is an association between adverse childhood experiences and adult psychopathology (Etain et al., 2010), an interesting hypothesis is that EMS may mediate this relationship, and thus develop and maintain BD (Ball et al., 2003). More generally, there is a kind of correspondance between BD severity and EMS activation; people with the least activated EMS are the ones who are doing the best in terms of BD severity, anxiety symptom intensity, remission duration, and psychosocial functioning.

Although previous studies have demonstrated associations between intelligence and personality traits (Bartels et al., 2012; Schaie et al., 2004; Berggren and Derakshan, 2013), our findings did not reveal a significant relationship between neuropsychological performance and EMS. This absence of association may reflect the specific neuropsychological dimensions assessed in this study, which could be complemented by measures of social cognition. Given the established link between EMS and interpersonal functioning (DeTore et al., 2018), social cognition might provide a more relevant framework for exploring this relationship. Furthermore, while cognitive functioning is a critical determinant of psychosocial functioning in BD (Roux et al., 2024; Roux et al., 2017; Ehrminger et al., 2021), its role appears distinct from that of EMS. Furthermore, cognitive complaints should be assessed because they are frequently reported by people with BD such as memory or attentional difficulties (Rosa et al., 2013) and reported as having an impact on their daily life. Our findings suggest that cognitive deficits do not necessarily hinder the implementation of schema therapy, as the presence of early maladaptive schemas (EMS) and cognitive abilities do not appear to be related. This study underscores the importance of distinguishing between cognitive functioning (e.g., memory, attention, executive function) and the content of cognitions (in this case, cognitive schemas). From a personalized care perspective, it is therefore essential to specifically evaluate the factors that hinder the patient. Combining functional remediation and schema therapy may prove effective for individuals with difficulties in both areas, as one approach cannot substitute for the other.

This study has several limitations. One limitation concerns the heterogeneity caused by the three subtypes of bipolar disorders investigated in this study. Understanding whether Bipolar I and Bipolar II disorders are the same or distinct conditions is a significant area of research. It would be interesting to replicate this study, specifically highlighting the results found for EMS in Bipolar I and Bipolar II separately. Moreover, the study did not control for the socioeconomic status and comorbid psychiatric disorders, which may influence EMS in BD. For example, several psychiatric disorders, which are common in BD, are associated with EMS, like attention deficit hyperactivity (Kiraz and Sertçelik, 2021), borderline personality (Frías et al., 2018), anxiety disorders (Koerner et al., 2015) and obsessive-compulsive disorders (Dostal and Pilkington, 2023). In this study, the role of socioeconomic status was not measured, yet it could be an influential factor in early maladaptive schemas (EMS) and psychosocial functioning. Especially since there is a link between social disadvantage (household income, education status, employment status) and functional impairment in people with bipolar disorder (Sylvia et al., 2017). Furthermore, this study was conducted with a French population. It would be interesting to replicate this study in other populations, both to test the effect of culture on the one hand, and the effect of certain demographic variables on the other hand. Additionally, the cross-sectional design of the study should be acknowledged as a limitation. Developing longitudinal designs could provide further valuable insights. Growth-mixture models could track EMS activation and functional outcomes over time. Then, if schema therapy interventions are tested, measuring changes in EMS factors or class membership can help confirm whether “Major Hyperactivation” patients particularly benefit.

## 5 Conclusion

In spite of its limitations, this study contributes to a more accurate understanding of the clinical heterogeneity of people in euthymic phases of BD. Clinicians should have an eco-systemic approach, taking into account environment, in particular childhood environment, and current environment. Initially developed to treat borderline personality disorders, other specific models of schema therapy have been developed to treat almost all other personality disorders (Csukly et al., 2011) and other disorders such as eating disorders, anxiety disorders and post-traumatic stress disorders (Taylor et al., 2017). Indeed, previous papers have argued the relevance of schema therapy as a treatment option for people with BD in the euthymic phase (Hawke et al., 2013; Ociskova et al., 2022).

## Data Availability

The original contributions presented in the study are included in the article/Supplementary material, further inquiries can be directed to the corresponding author.

## Footnotes

^1^ www.fondation-fondamental.org
