# Supplementary material for: Discrete early maladaptive schema subgroups in remitted bipolar disorders: association with neuropsychological performance, residual symptoms, and psychosocial functioning
Source: Front Psychol. 2025 Mar 18;16:1476345. doi: 10.3389/fpsyg.2025.1476345 (PMC11958715; doi:10.3389/fpsyg.2025.1476345)
Supplement: Supplementary file 1 [file Data_Sheet_1.docx]

**Supplementary information**. Results of the multiple regression analysis

Multiple regression of marital status on the type of cluster with all clinical and demographic covariates selected in the univariate analyses:

| **term** | **estimate** | **std.error** | **statistic** | **df** | **p.value** | **fmi** | **lambda** |
| --- | --- | --- | --- | --- | --- | --- | --- |
| Age (years) | 0.1 | 0.03 | 3.3 | 85.9 | 0.002 | 0.08 | 0.06 |
| Bipolar disorder type 1 | -1.85 | 1.24 | -1.5 | 84.6 | 0.139 | 0.09 | 0.07 |
| Bipolar disorder type 2 | -0.7 | 1.07 | -0.7 | 90.3 | 0.513 | 0.04 | 0.02 |
| Undetermined valence | 0.7 | 0.77 | 0.9 | 71.8 | 0.367 | 0.2 | 0.18 |
| Predominant manic valence | 1.09 | 1.18 | 0.9 | 52.2 | 0.36 | 0.37 | 0.35 |
| Age of onset (years) | -0.02 | 0.04 | -0.6 | 87 | 0.559 | 0.07 | 0.05 |
| CGI (Clinical Global Impression scale) | 0.06 | 0.38 | 0.2 | 84.3 | 0.873 | 0.1 | 0.08 |
| MADRS | -0.01 | 0.09 | -0.1 | 89.2 | 0.933 | 0.05 | 0.03 |
| End of last episode > 3 months | -0.86 | 0.68 | -1.3 | 84.5 | 0.205 | 0.09 | 0.07 |
| Lithium | -0.33 | 0.8 | -0.4 | 85.4 | 0.681 | 0.09 | 0.07 |
| Antipsychotic | -0.67 | 0.81 | -0.8 | 85.9 | 0.412 | 0.08 | 0.06 |
| CTQ | 0.07 | 0.02 | 2.8 | 87.3 | 0.007 | 0.07 | 0.05 |
| STAY-A | 0.01 | 0.03 | 0.3 | 90.9 | 0.743 | 0.03 | 0.01 |
| Substance Use Disorder (lifetime) | -0.29 | 0.77 | -0.4 | 82 | 0.711 | 0.12 | 0.1 |
| EMS cluster (Light Hyperactivation vs Hypoactivation) | -2.21 | 0.72 | -3.1 | 88.6 | 0.005 | 0.06 | 0.04 |
| EMS cluster Major Hyperactivation vs Hypoactivation | -3.76 | 1.15 | -3.3 | 84.5 | 0.005 | 0.09 | 0.07 |
| EMS cluster Major Hyperactivation vs Light Hyperactivation | -1.55 | 0.86 | -1.8 | 85.4 | 0.075 | 0.09 | 0.07 |

**Term** (specific component or category of the variable being tested); **Estimate** (estimated value of the regression coefficient); Std.error (Standard Error); df (Degrees of Freedom); fmi (Fraction of Missing Information) CGI (Clinical Global Impression scale); MADRS (Montgomery-Åsberg Depression Rating Scale); CTQ (Childhood Trauma Questionnaire): childhood trauma score; STAI forme YA (State Anxiety Inventory forme YA), EMS: Early Maladaptive Scheme

Multiple regression of Functioning Assessment Short Test (FAST) on the type of cluster with all clinical and demographic covariates selected in the univariate analyses

| **term** | **estimate** | **std.error** | **statistic** | **df** | **p.value** | **fmi** | **lambda** |
| --- | --- | --- | --- | --- | --- | --- | --- |
| Age (years) | 0.07 | 0.09 | 0.8 | 90.8 | 0.423 | 0.03 | 0.01 |
| Bipolar disorder type 1 | 0.83 | 3.46 | 0.2 | 90.3 | 0.811 | 0.04 | 0.02 |
| Bipolar disorder type 2 | -2.83 | 3.16 | -0.9 | 91.3 | 0.373 | 0.03 | 0.01 |
| Undetermined valence | 1.84 | 2.38 | 0.8 | 79.5 | 0.442 | 0.14 | 0.12 |
| Predominant manic valence | 0.98 | 3.19 | 0.3 | 75.9 | 0.76 | 0.17 | 0.15 |
| Age of onset (years) | -0.15 | 0.14 | -1.1 | 86.8 | 0.273 | 0.07 | 0.05 |
| CGI (Clinical Global Impression scale) | 0.99 | 1.3 | 0.8 | 91 | 0.448 | 0.03 | 0.01 |
| MADRS | 0.59 | 0.32 | 1.8 | 90.4 | 0.068 | 0.04 | 0.02 |
| End of last episode > 3 months | -7.35 | 2.07 | -3.6 | 89.9 | 0.001 | 0.04 | 0.02 |
| Lithium | -0.18 | 2.6 | -0.1 | 89.2 | 0.944 | 0.05 | 0.03 |
| Antipsychotic | 0.96 | 2.48 | 0.4 | 90.8 | 0.701 | 0.03 | 0.01 |
| CTQ | -0.01 | 0.07 | -0.2 | 90.4 | 0.864 | 0.04 | 0.02 |
| STAY-A | 0.19 | 0.1 | 1.9 | 90.4 | 0.056 | 0.04 | 0.02 |
| Substance Use Disorder (lifetime) | -1.8 | 2.62 | -0.7 | 87 | 0.493 | 0.07 | 0.05 |
| EMS cluster (Light Hyperactivation vs Hypoactivation) | 5.17 | 2.2 | 2.4 | 90 | 0.063 | 0.04 | 0.02 |
| EMS cluster Major Hyperactivation vs Hypoactivation | 5.08 | 3.22 | 1.6 | 90.4 | 0.177 | 0.04 | 0.02 |
| EMS cluster Major Hyperactivation vs Light Hyperactivation | -0.09 | 2.85 | 0 | 90 | 0.974 | 0.04 | 0.02 |

**Term** (specific component or category of the variable being tested); **Estimate** (estimated value of the regression coefficient); Std.error (Standard Error); df (Degrees of Freedom); fmi (Fraction of Missing Information) CGI (Clinical Global Impression scale); MADRS (Montgomery-Åsberg Depression Rating Scale); CTQ (Childhood Trauma Questionnaire): childhood trauma score; STAI forme YA (State Anxiety Inventory forme YA).

`

Multiple regression of Global Assessment of Functioning (GAF) on the type of cluster with all clinical and demographic covariates selected in the univariate analyses:

| **term** | **estimate** | **std.error** | **statistic** | **df** | **p.value** | **fmi** | **lambda** |
| --- | --- | --- | --- | --- | --- | --- | --- |
| Age (years) | -0.08 | 0.11 | -0.7 | 90.4 | 0.483 | 0.04 | 0.02 |
| Bipolar disorder type 1 | -1.39 | 4.33 | -0.3 | 88.8 | 0.748 | 0.05 | 0.03 |
| Bipolar disorder type 2 | 1.84 | 3.91 | 0.5 | 91.6 | 0.639 | 0.03 | 0.01 |
| Undetermined valence | -1.84 | 2.98 | -0.6 | 77.6 | 0.538 | 0.15 | 0.13 |
| Predominant manic valence | 0.22 | 4.23 | 0.1 | 62.7 | 0.96 | 0.28 | 0.26 |
| Age of onset (years) | 0.1 | 0.17 | 0.6 | 87.6 | 0.559 | 0.07 | 0.05 |
| CGI (Clinical Global Impression scale) | -1.8 | 1.63 | -1.1 | 89.8 | 0.27 | 0.04 | 0.02 |
| MADRS | -0.44 | 0.39 | -1.1 | 90.3 | 0.266 | 0.04 | 0.02 |
| End of last episode > 3 months | 5.56 | 2.55 | 2.2 | 91 | 0.032 | 0.03 | 0.01 |
| Lithium | 0.4 | 3.21 | 0.1 | 90.1 | 0.902 | 0.04 | 0.02 |
| Antipsychotic | -0.27 | 3.07 | -0.1 | 90.8 | 0.93 | 0.03 | 0.01 |
| CTQ | 0.03 | 0.09 | 0.4 | 90.2 | 0.698 | 0.04 | 0.02 |
| STAY-A | -0.26 | 0.12 | -2.1 | 90.9 | 0.039 | 0.03 | 0.01 |
| Substance Use Disorder (lifetime) | 2.25 | 3.38 | 0.7 | 78.1 | 0.508 | 0.15 | 0.13 |
| EMS cluster (Light Hyperactivation vs Hypoactivation) | -1.76 | 2.71 | -0.6 | 90.5 | 0.518 | 0.04 | 0.02 |
| EMS cluster Major Hyperactivation vs Hypoactivation | -6.22 | 4.02 | -1.5 | 89.2 | 0.318 | 0.05 | 0.03 |
| EMS cluster Major Hyperactivation vs Light Hyperactivation | -4.46 | 3.54 | -1.3 | 89.2 | 0.318 | 0.05 | 0.03 |

**Term** (specific component or category of the variable being tested); **Estimate** (estimated value of the regression coefficient); Std.error (Standard Error); df (Degrees of Freedom); fmi (Fraction of Missing Information) CGI (Clinical Global Impression scale); MADRS (Montgomery-Åsberg Depression Rating Scale); CTQ (Childhood Trauma Questionnaire): childhood trauma score; STAI forme YA (State Anxiety Inventory forme YA).

`

Multiple regression of quality of life (EQ5D) on the type of cluster with all clinical and demographic covariates selected in the univariate analyses

| **term** | **estimate** | **std error** | **statistic** | **df** | **p.value** | **fmi** | **lambda** |
| --- | --- | --- | --- | --- | --- | --- | --- |
| Age (years) | 0 | 0 | -0.7 | 79.9 | 0.459 | 0.13 | 0.11 |
| Bipolar disorder type 1 | 0.06 | 0.06 | 1 | 66 | 0.327 | 0.25 | 0.23 |
| Bipolar disorder type 2 | 0.09 | 0.05 | 1.9 | 74.6 | 0.064 | 0.18 | 0.16 |
| Undetermined valence | -0.03 | 0.04 | -0.7 | 41.8 | 0.458 | 0.48 | 0.45 |
| Predominant manic valence | 0.04 | 0.05 | 0.7 | 58.8 | 0.472 | 0.31 | 0.29 |
| Age of onset (years) | 0 | 0 | 0.8 | 59.9 | 0.426 | 0.3 | 0.28 |
| CGI (Clinical Global Impression scale) | 0 | 0.02 | 0.2 | 76.3 | 0.852 | 0.17 | 0.14 |
| MADRS | 0 | 0.01 | -0.5 | 66.1 | 0.626 | 0.25 | 0.23 |
| End of last episode > 3 months | 0.05 | 0.04 | 1.5 | 60.2 | 0.128 | 0.3 | 0.28 |
| Lithium | -0.08 | 0.04 | -2 | 72.1 | 0.049 | 0.2 | 0.18 |
| Antipsychotic | -0.04 | 0.04 | -1.1 | 85.8 | 0.276 | 0.08 | 0.06 |
| CTQ | 0 | 0 | -1.8 | 57.2 | 0.082 | 0.33 | 0.3 |
| STAY-A | -0.01 | 0 | -4.5 | 65 | <0.001 | 0.26 | 0.24 |
| Substance Use Disorder (lifetime) | -0.02 | 0.05 | -0.5 | 52 | 0.643 | 0.37 | 0.35 |
| EMS cluster Light Hyperactivation vs Hypoactivation | -0.03 | 0.04 | -0.8 | 66.1 | 0.78 | 0.25 | 0.23 |
| EMS cluster Major Hyperactivation vs Hypoactivation | -0.02 | 0.06 | -0.3 | 59.3 | 0.78 | 0.31 | 0.29 |
| EMS cluster Major Hyperactivation vs Light Hyperactivation | 0.01 | 0.05 | 0.3 | 60.3 | 0.78 | 0.3 | 0.28 |

**Term** (specific component or category of the variable being tested); **Estimate** (estimated value of the regression coefficient); Std.error (Standard Error); df (Degrees of Freedom); fmi (Fraction of Missing Information) CGI (Clinical Global Impression scale); MADRS (Montgomery-Åsberg Depression Rating Scale); CTQ (Childhood Trauma Questionnaire): childhood trauma score; STAI forme YA (State Anxiety Inventory forme YA).

`
